# Clonogenicity: Holoclones and Meroclones Contain Stem Cells

**DOI:** 10.1371/journal.pone.0089834

**Published:** 2014-02-26

**Authors:** Charlotte M. Beaver, Aamir Ahmed, John R. Masters

**Affiliations:** Prostate Cancer Research Centre, University College London, London, United Kingdom; Southern Illinois University School of Medicine, United States of America

## Abstract

When primary cultures of normal cells are cloned, three types of colony grow, called holoclones, meroclones and paraclones. These colonies are believed to be derived from stem cells, transit-amplifying cells and differentiated cells respectively. More recently, this approach has been extended to cancer cell lines. However, we observed that meroclones from the prostate cancer cell line DU145 produce holoclones, a paradoxical observation as meroclones are thought to be derived from transit-amplifying cells. The purpose of this study was to confirm this observation and determine if both holoclones and meroclones from cancer cell lines contain stem cells. We demonstrated that both holoclones and meroclones can be serially passaged indefinitely, are highly proliferative, can self-renew to form spheres, are serially tumorigenic and express stem cell markers. This study demonstrates that the major difference between holoclones and meroclones derived from a cancer cell line is the proportion of stem cells within each colony, not the presence or absence of stem cells. These findings may reflect the properties of cancer as opposed to normal cells, perhaps indicating that the hierarchy of stem cells is more extensive in cancer.

## Introduction

The relationship between stem cell capacity and colony forming ability of primary keratinocytes was established in a seminal paper by Barrandon and Green [1]. Using primary cultures of human keratinocytes, Barrandon and Green found that single cells produced 3 types of colony (which they termed holoclones, meroclones and paraclones) derived from cells with different proliferative capacities. Only holoclones are capable of extensive proliferation and self-renewal, whilst meroclones have a limited proliferative capacity and cannot self-renew and paraclones are incapable of further proliferation. The terms holoclone, meroclone and paraclone have since become synonymous with colonies derived respectively from stem, early and late stage transit-amplifying cells [1], [2].

The hierarchy of colony forming cells described by Barrandon and Green is recapitulated by certain types of normal cell in culture [3], [4] and consequently colony morphology is used routinely as a surrogate assay to identify and characterize stem cells from skin [5], [6], follicular [7] and limbal [8], [9] tissues. The assay is also used to evaluate stem cells for use in tissue engineering [6]. Holoclones express survival and self-renewal genes associated with stem cell capacity, such as p63 [8] and BMI-1 [10]. In these studies, the colonies and not the individual cell they are derived from are referred to as holoclones, meroclones and paraclones.

Subsequently, holoclones, meroclones and paraclones were described in clones derived from human cancer cell lines of various types, including pancreatic [11], head and neck [12], breast [13] and prostate [14]–[18]. Cancer cell holoclones can be passaged indefinitely [14] and xenografted serially [17]. The formation of holoclones has been adopted as a surrogate stem cell assay, particularly in the study of prostate cancer [19]–[22]. An increased number of holoclones is regarded as enrichment for cancer stem cells (CSC) and has been used to study CSC marker expression. In prostate cancer an increased number of holoclones is associated with the expression of the putative stem cell markers CD44, integrin α2β1, CD133 [14], [17], PSA^lo^ expression [23] and aldehyde dehydrogenase 1 (ALDH) activity [20]. Holoclone formation has also been used to validate sphere formation from cell lines as a stem cell assay [24] and to demonstrate the presence of cancer stem cells in samples from primary human prostate cancers [25]. In addition, holoclone formation has been used to demonstrate enrichment of cancer stem cells in side population ovarian cancer cells [26] and in CD133 [27] and [12] CD44 expressing oral squamous cancer cell lines.

We set out to use the colony forming assay as a surrogate marker to identify genes that control self-renewal in prostate cancer cells. However, we observed that colonies derived from meroclones (putatively derived from transit-amplifying cells) were able to produce holoclones (stem cell colonies), albeit at a lower frequency than the colonies derived from holoclones. This observation calls into question the widely held and applied assumption that colonies with the three characteristic morphologies are derived from stem, early and late transit-amplifying cells respectively.

We therefore set out to re-investigate the relationship between clonogenicity and stem cell capacity in cancer cells by studying the colony forming ability, transplantation capacity and marker expression of each morphological type of colony derived from the prostate cancer cell line DU145. We tested the hypothesis that the cancer cell colonies differ in the proportion, rather than the presence or absence, of stem cells. The results support this hypothesis. The experiments did not test the original findings of Barrandon and Green, which were based on normal cells, and consequently may indicate that self-renewal capacity is extended further down the stem cell hierarchy in cancer.

## Materials and Methods

### Cell Culture

The prostate cancer cell line DU145 was obtained from its originator [28] and maintained in 25 cm^2^ culture flasks containing growth medium RMPI-1640 (Invitrogen, UK) supplemented with 10% FBS (PAA, UK) and 2 mM L-Glutamine (Invitrogen) at 37°C in 5% CO_2_. A single cell suspension was prepared by incubating cells with 0.25% trypsin for 10 min and counted using trypan blue exclusion. To determine colony forming efficiency and for serial cloning, 200 single cells were seeded into 60 mm diameter petri-dishes with 5 ml growth medium and incubated for two weeks until macroscopic colonies were visible. To check the proportion of colonies that arise from single cells, colony growth was monitored by the Incucyte Live Cell Imaging System from initial adherence every 4 hours for 2 weeks.

### Colony Analysis

Following 2 weeks incubation, dishes were fixed by removal of culture medium and the addition of 70% IMS. Colonies were stained with 0.1% crystal violet (Sigma-Aldrich) and the number and type of colonies counted. Total CFE and the CFE of each colony type was calculated as a percentage of the number of cells seeded. Using a graticule and eye piece, the colony was measured across perpendicular axes to estimate the area of each colony. The number of cells across the diameter was counted and thus the area per cell and the total cell number per colony were estimated. To determine average colony dimensions, 20 colonies of each type were measured and the number of cells in each colony calculated.

### Secondary Cloning

Colonies were inspected under a light microscope and scored on morphology and the size and number of cells per colony was determined as described above. Fifteen well isolated colonies of each type were selected and ring cloned by placing a glass cloning ring around the colony sealed with vacuum grease and trypisinised by adding 75 µl trypsin for 5 min. The colony was resuspended in medium, transferred to fresh 60 mm diameter petri dishes at a density of 200 cells per dish and incubated for a further 2 weeks. Dishes were fixed and stained with crystal violet and the CFE determined.

### Serial Cloning

In three separate experiments, three colonies of each type were serially cloned for up to 10 passages. Colonies were ring cloned for secondary cloning as described above and resultant colonies were then serially cloned using the same method. Each lineage was serially cloned up to 10 times or until the colony was terminal. As meroclone were able to form secondary holoclones, these secondary holoclones were also serially cloned. Remaining dishes at each generation were fixed with 70% IMS and stained with 0.1% crystal violet (Sigma-Aldrich) for analysis of CFE and colony morphology.

### Serial Passage (Bulk)

Fifteen colonies of each type were ring cloned as described above and transferred to a 25 cm^2^ flask containing culture medium. Each clone was incubated until 70–90% confluent and passaged 1 in 6 for up to 20 passages. Clones that failed survive to reach confluency were monitored for the duration of the experiment, until all surviving clones reached 20 passages.

### Sphere Forming Assay

Ring cloned DU145 colonies were seeded at a density of 1000 cells per well of 6 well plate in 135 µl sphere culture medium (serum-free DMEM/F12 medium, 20 ng/ml basic FGF, 20 ng/ml EGF 1x B27 (all Invitrogen) and 3 µg/ml insulin (Sigma)) mixed 1∶1 with 135 µl Matrigel Basement Membrane Matrix (BD Bioscience, Michigan, USA) and pipetted gently around the edges of a 6 well plate (Nunc). The plate was incubated at 37°C for 15 min until the Matrigel had set. It was covered with 3 ml sphere culture medium and incubated for 2 weeks with a 50% medium change at 7 days. Following 14 days incubation, spheres were counted and their diameter measured using an eye piece and graticule to determine sphere forming efficiency (% SFE) and sphere size.

### Xenograft Tumor Formation

This study was performed in accordance with the recommendations in the *Guidelines for the welfare and use of animals in cancer research* and every effort was made to minimize suffering [29]. The work was carried out under the authority of Home Office, UK, approved project licence (PPL 70/7244). Holoclones and meroclones were harvested, pooled. 10,000 or 1,000 cells were resuspended 1∶1 in RPMI/Matrigel mixture and injected subcutaneously into the flanks of 7 week old male Nude mice (Harlan, UK). Mice were inspected for tumor growth by palpation and tumor growth was measured weekly using a digital caliper (WPI, Florida, USA). Tumor growth was determined using the formula a×b^2^×0.5, where a is the longer and b the shorter of the two perpendicular diameters. Mice were killed by cervical dislocation at 12 weeks and tumors removed and weighed.

Tumors were minced into ∼1 mm^3^ pieces in RPMI-1640 containing 10% FBS and 100 U/mL Penicillin/streptomycin (Invitrogen) and washed twice with the same medium and then digested in 10 ml/g tissue of 600 Uml collagenase (Invitrogen) for 2 hours at 37°C with gentle shaking. The resulting cell suspension was washed and a single cell suspension obtained by filtering through a 40 µm cell strainer. The single cell suspension was used to measure clonogenicity and for serial xenotransplantation.

### Fluorescence Immunocytochemistry

Colonies grown at a density of 10 cells per well in 24 well plates for 2 weeks were fixed with 4% PFA for 20 min at room temperature. Colonies stained for intracellular markers (Ki67, K5, K18, BMI-1 and Oct4) were permeabilised by adding 200 µl 0.2% Triton X-100 (Sigma-Aldrich) in PBS for 10 min at room temperature. Non-specific staining was blocked by incubating the colonies for 30 min with 10% normal goat serum (NGS) (PAA) at room temperature. Primary antibodies (Table S1) (Abcam, UK) diluted in 10% NGS in PBS were incubated overnight at 4°C, washed 4 times and incubated with FITC-conjugated secondary antibodies (Southern Biotech) for 1 h at room temperature. Finally, the colonies were washed, dried and mounted using Vectashield containing DAPI (Vector Laboratories Inc, Peterborough, U.K.). Colonies were viewed using Olympus Total Internal Reflection inverted confocal microscope and Fluoview 2000 software. The marker positive fraction was calculated as a percentage of total cell number (number of DAPI stained nuclei).

### Statistical Analysis

The properties of each colony type were analysed by one way analysis of variance (ANOVA) or Multivariate analysis of variance (MANOVA) followed by Tukey’s honestly significant difference (HSD) post hoc pairwise comparison using the statistics package PAWS Statistics 18 (formerly SPSS). Results displayed as percentages were analysed following arcsine transformation. p values of less than 0.05 were deemed significant.

## Results

**Figure 1:**
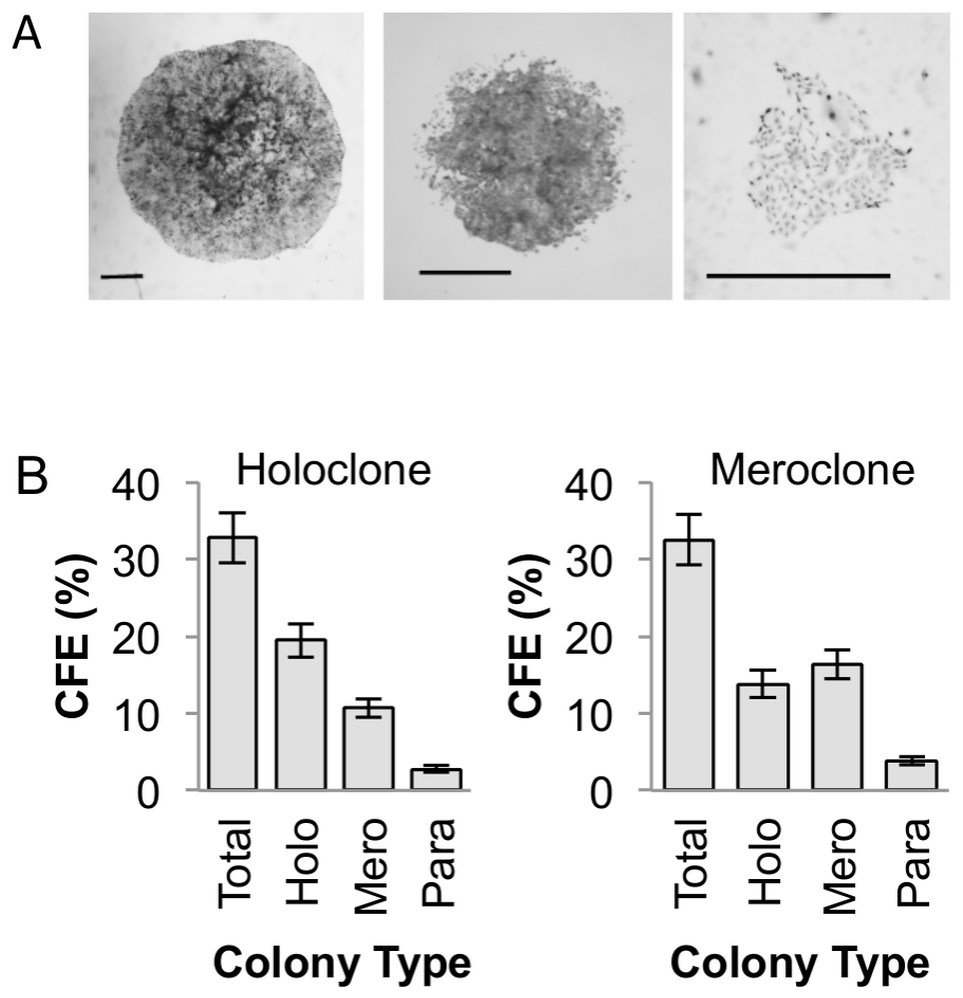
Clonogenicity of DU145 prostate cancer cell line. (A) Single DU145 cells form colonies of three morphological types termed holo-, mero-, and paraclones (pictured) Bar = 200 µm. (B) Type 1 (left) and type 2 (right) colonies which were ring cloned and cultured at clonal density formed secondary colonies. The secondary colony forming efficiency (CFE) and types of secondary colonies were determined. Paraclone were unable to from secondary colonies.

DU145 cells form three types of colony (Figure 1A), with morphologies characteristic of holoclones, meroclones and paraclones [18] The number of cells in each colony was estimated by measuring the diameters of twenty colonies of each type and determining the number of cells across the diameter. Holoclones are large with smooth edges and consist of small tightly packed cells with a mean density of 1470±400 cells/cm^2^. Meroclones are smaller, have an irregular outline and consist of a mixture of small tightly packed cells and much larger loosely packed cells, particularly around the edge, with an average cell density of 753±218 cells/cm^2^. Paraclones are small and diffuse and consist mainly of loosely packed enlarged cells with a mean density of 261±74 cells/cm^2^.

### Colony Forming Efficiency (CFE)

In order to measure secondary CFE, 30 colonies of each type were cloned and plated at 200 cells/6 cm dish in triplicate and the numbers of each type of colony produced were counted (Figure 1B). Holoclones and meroclones produced similar numbers of secondary colonies overall, whereas paraclones produced few or no secondary colonies. The major difference between holoclones and meroclones was the number of secondary holoclones produced. Holoclones produced mainly holoclones, whereas meroclone colonies produced slightly more meroclones than holoclones. The production of holoclones from meroclones is paradoxical as this finding would suggest that colonies derived from transit-amplifying cells can produce stem cell colonies. Therefore we tested the hypothesis that holoclones and meroclones differ only in the proportion of stem cells.

### Can Each Type of Colony be Serially Cloned?

To determine if both holoclones and meroclones have indefinite proliferative capacity, the serial colony forming capacity of each colony type was measured. 3 holoclones and 3 meroclones were picked, and re-cloned at 200 cells/5 cm dish in triplicate. Holoclones from holoclones and meroclones from meroclones were serially cloned up to 17 times as for the first cloning. Additionally, holoclones derived from original meroclones following the first round of cloning were also serially cloned a further 10 times in the same way. Based on the estimate of the number of cells in each colony, it was possible to calculate the number of cell divisions needed to produce each colony. This calculation was a crude estimate as it assumed no cell loss and identical reproductive capacity throughout the colony (Table 1). Over the course of the experiment, it was calculated that the holoclones derived from holoclones had undergone a minimum of 129 cell divisions, holoclones derived from meroclones 117 cell divisions, whereas the meroclones derived from meroclones died out after 66 cell divisions. Both the holoclones derived from holoclones and meroclones continued to produce further holoclones for the duration of the experiment, whereas the serial meroclones continued to produce holoclones for only 4 rounds and died out after 7 rounds of cloning.

**Table 1:** Number of Cell Divisions during Serial Cloning.

Generation	Holoclone	Holoclone from Meroclone	Meroclone
1	3222 (11)	1650 (10)	1650 (10)
2	4385 (12)	2752 (11)	801 (9)
3	11700 (13)	3728 (11)	1104 (10)
4	3320 (11)	5344 (12)	1364 (10)
5	7695 (12)	3029 (11)	668 (9)
6	3521 (11)	2843 (11)	947 (9)
7	5470 (12)	1849 (10)	636 (9)
8	3029 (11)	1923 (10)	−
9	7847 (12)	2237 (11)	−
10	3728 (11)	665 (9)	−
11	12693 (13)	3222 (11)	−
**Total Number**	**129**	**117**	**66**

Each colony type was serially cloned and the proliferative capacity of each clone determined. Colony size was used to estimate the number of cell divisions at each generation displayed as mean cell number and minimum number of cell divisions in brackets. The sum of divisions at each generation provides an estimate of how many total cell divisions the original cell which formed the original colony of each type can undergo.

### Can Each Type of Colony be Serially Passaged?

In order to study self-renewal capacity using an alternative method, 5 holoclones and 5 meroclones were ring-cloned and transferred in bulk to T25 flasks. The experiment was repeated 3 times. 14/15 (93%) of holoclone and 10/15 (67%) meroclones were still growing at the same rate after 20 passages. Initially, the growth rate of holoclones was faster than that of meroclones. However, after 4 weeks, the growth rates of cells derived from the two colony types were similar and each clone showed the typical morphology of DU145 cells grown in a monolayer.

### Can Each Type of Colony form Spheres?

The sphere-forming assay is used as a measure of self-renewal. In three experiments, 3 of each type of colony were harvested and plated at 1000 cells/well in a 6 well plate in triplicate. Holoclones had a sphere forming efficiency of 15.3% ±3.1% compared to 5.9% ±2.7% for meroclones (p<0.05, one-way ANOVA). The spheres formed by holoclones were larger than those of meroclones with a mean diameter of 105 µm ±5.1 µm compared to 63 µm ±6.3 µm. Paraclones were unable to form spheres.

### Tumorigenicity

Colonies of each type were transplanted subcutaneously into the flanks of nude mice. The data for holoclones were based on individual colonies, whereas those for meroclones and paraclones were based in part on pooling colonies to provide the number of cells required (Table 2).

**Table 2:** Tumorigenicity of DU145 colonies.

Cell Type	Number of Cells Injected	Tumor Incidence	Latency (d)
**DU145 Monolayer**	10000	5/6	37.8 (11)
	1000	4/8	41.8 (9)
**Holoclone**	10000	4/9	30.5 (8)
	1000	4/12	49.5 (13)
**Meroclone**	10000	2/7	47 (4)
	1000	4/16	61.5 (3)
**Paraclone**	1000	0/4	−
**Vehicle Control**	0	0/12	−

DU145 colonies were pooled and were injected s.c into the flanks of Nude mice in a mixture of 1∶1 Matrigel: RPMI. Vehicle control (VC) animals received and injection of Matrigel: RPMI alone. Tumor latency was determined as the first day tumours were palpable and animals sacrificed after 12 weeks and tumors weighed. Mean latency and tumor weights display, standard error in brackets.

**Figure 2:**
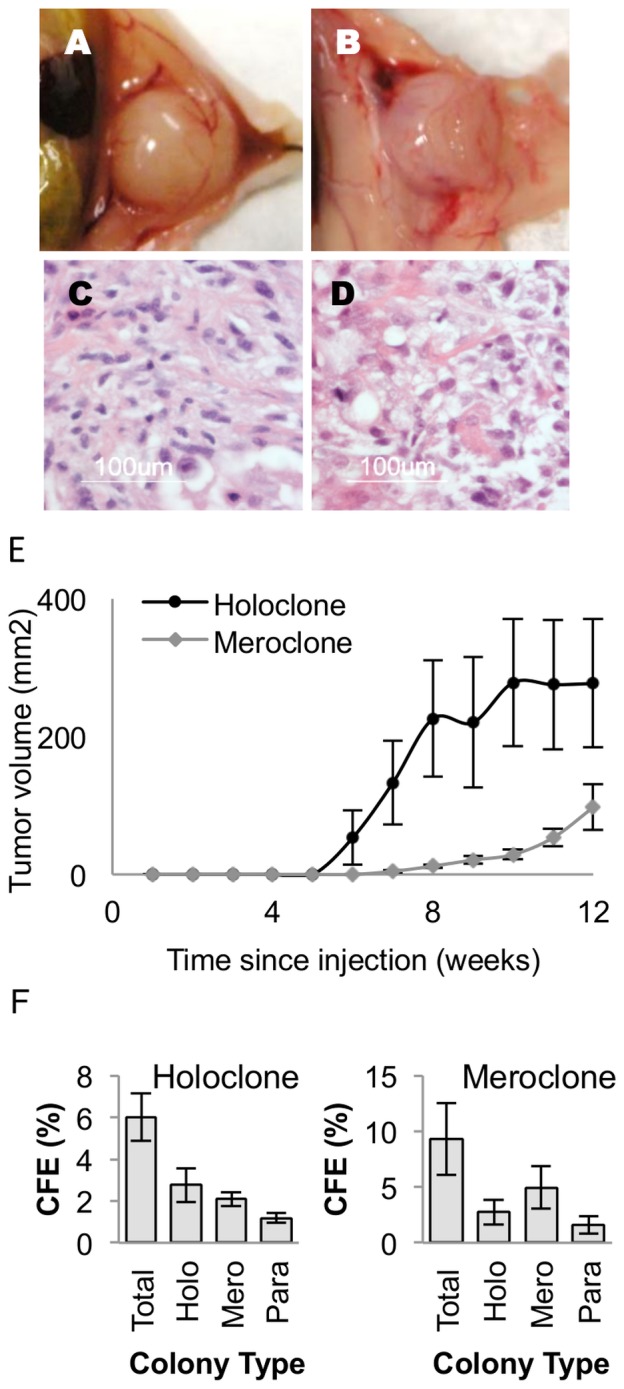
Tumourigenicity of DU145 colonies. DU145 colonies of each type were injected subcutaneously into the flanks of nude mice. Holoclones (A) and Meroclones (B) both formed tumours which were excised and stained with H&E (C&D). (E) The tumours derived from 10000 cells were measured by digital callipers across 2 diameters at 180° weekly and tumor volume calculated (mean ± S.E.). (F) Clonogenicity of tumors. Following dissection, tumors were digested in collagenase to produce a single cell suspension. 200 cells were seeded into petri dishes to determine colony forming efficiency (%) and the types of colonies formed by tumors of parent colonies.

Holoclones and meroclones, but not paraclones, were able to initiate tumor development in nude mice (Figure 2A–D). There was little difference between holoclones and meroclones in their ability to develop cancers, but meroclones had a longer latency and the tumours did not grow as large over the 12 week period as those derived from holoclones (Figure 2E). Cells isolated from the transplanted tumors and cultured in vitro in a clonogenic assay recapitulated the three colony types in similar proportions to the original cell line and could be xenografted (Figure 2F).

### Does the Proliferative Fraction of the Three Colony Types Differ?

**Figure 3:**
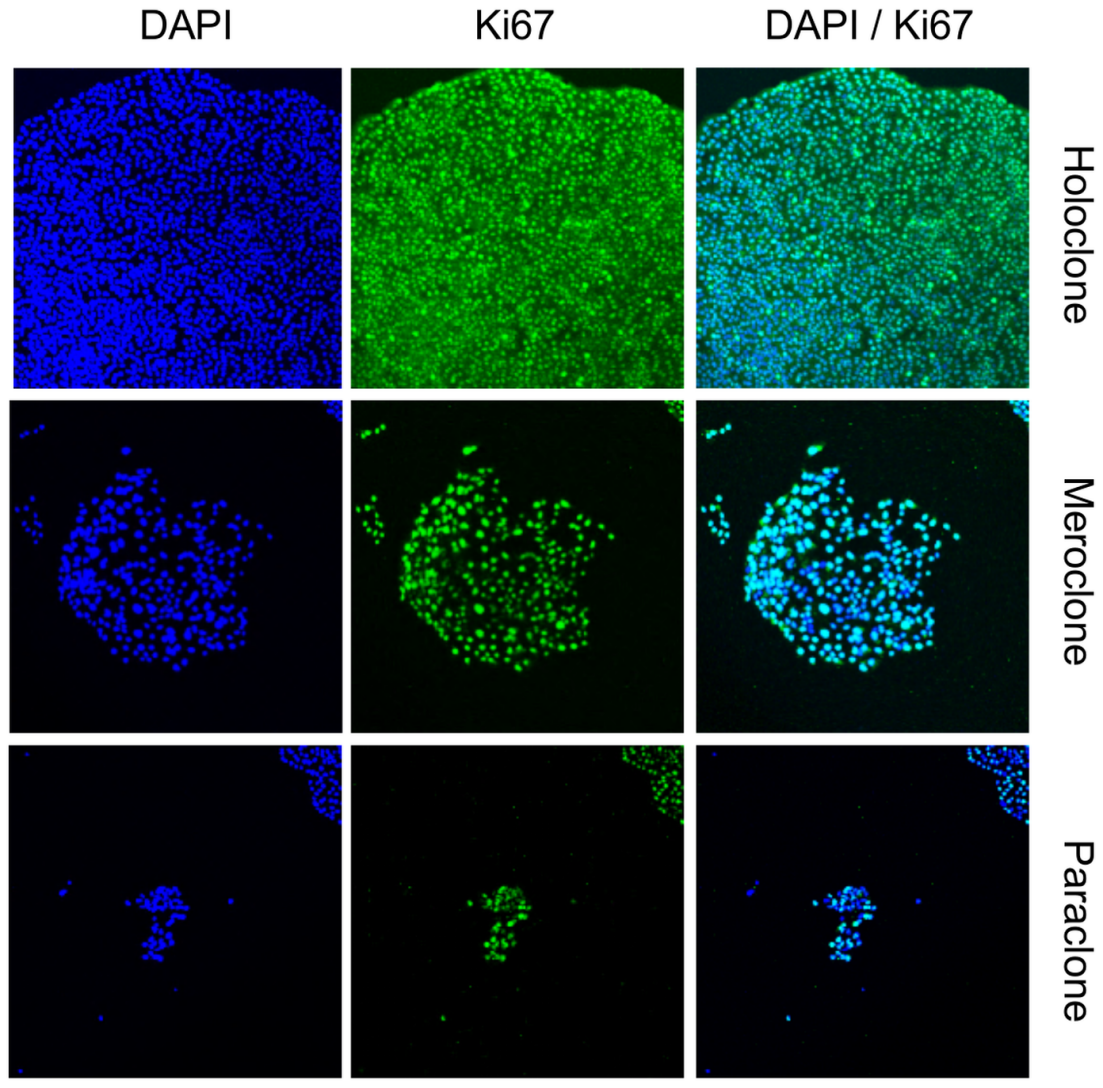
The proliferative fraction of DU145 colonies types determined by Ki67 staining. The percentage of Ki67 positive cells was determined by counting the number of green (FITC) cells as a proportion of blue DAPI positive nuclei. Representative colonies shown.

From three experiments, a total of 20 colonies of each type were fixed in paraformaldehyde and stained for Ki-67. The majority of the cells in all 3 types of colony were Ki-67 positive (Figure 3), with 86.6±3.6% positive in holoclones, 71.7±3.3% in meroclones and 58.9±7.5% in paraclones. Ki-67 expression was spread evenly throughout the colonies.

### Do the Colonies Differ in Marker Expression?

From three experiments a total of 20 colonies of each type growing in 24 well plates were fixed in paraformaldehyde and stained for each marker.

**Figure 4:**
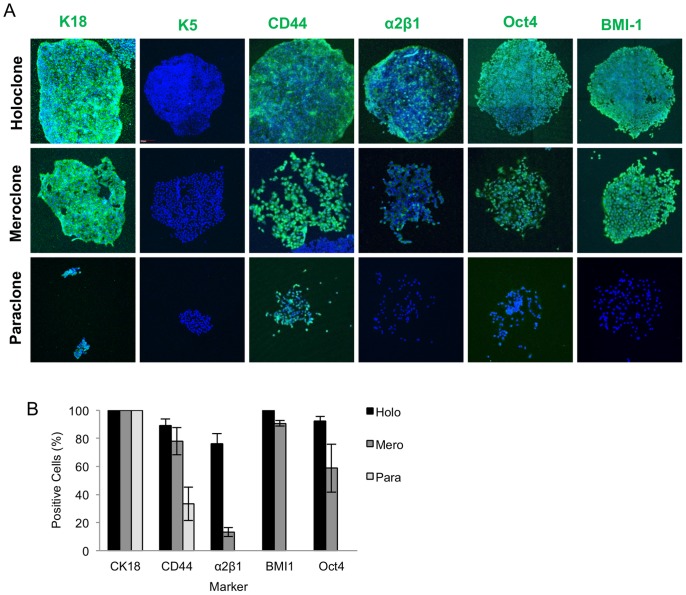
Expression of epithelial and stem cell markers by DU145 colonies. Expression of luminal (K18) and basal (K5) epithelial and stem cell markers (CD44, α2β1 integrin, Oct4 and BMI1) in DU145 colonies was determined by immunocytochemistry. (A) Holo, mero and paraclone DU145 colonies were stained by immunocytochemistry with monoclonal antibodies against the target, detected with a FITC conjugated secondary antibody (Green) and counter stained with DAPI (blue). (B) The number of positive cells for each maker were determined as a percentage of the total number of cells counted. Holoclone and meroclones contained more CD44 positive cells than paraclones. Hololcones colonies contained more α2β1 positive cells than meroclones. p<0.05 (MANOVA).

Cytokeratin 18 (K18) is characteristic of prostate epithelial luminal cells, while cytokeratin 5 (K5) is characteristic of the less differentiated basal cells. All the cells in all three colony types expressed K18, but no cells expressed K5 (Figure 4).

Colonies were stained for 4 markers for which there is evidence of association with epithelial stem cells in the human prostate, namely CD44, α2β1 integrin, Oct4 and BMI-1. Over 80% of the cells in holoclones stained for all the markers. The intensity of CD44 and α2β1 integrin was variable, with more intensely staining cells tending to be located around the periphery of the colony. Meroclones had a similar number of Oct4, BMI-1 and α2β1 integrin positive cells as holoclones, but contained fewer α2β1 integrin cells (figure 4 B). Paraclones were negative for α2β1 integrin, BMI-1 and Oct-4 and contained fewer CD44 positive cells than the holoclones and meroclones (p<0.05 MANOVA).

### Are Colonies Derived from Single Cells?

**Figure 5:**
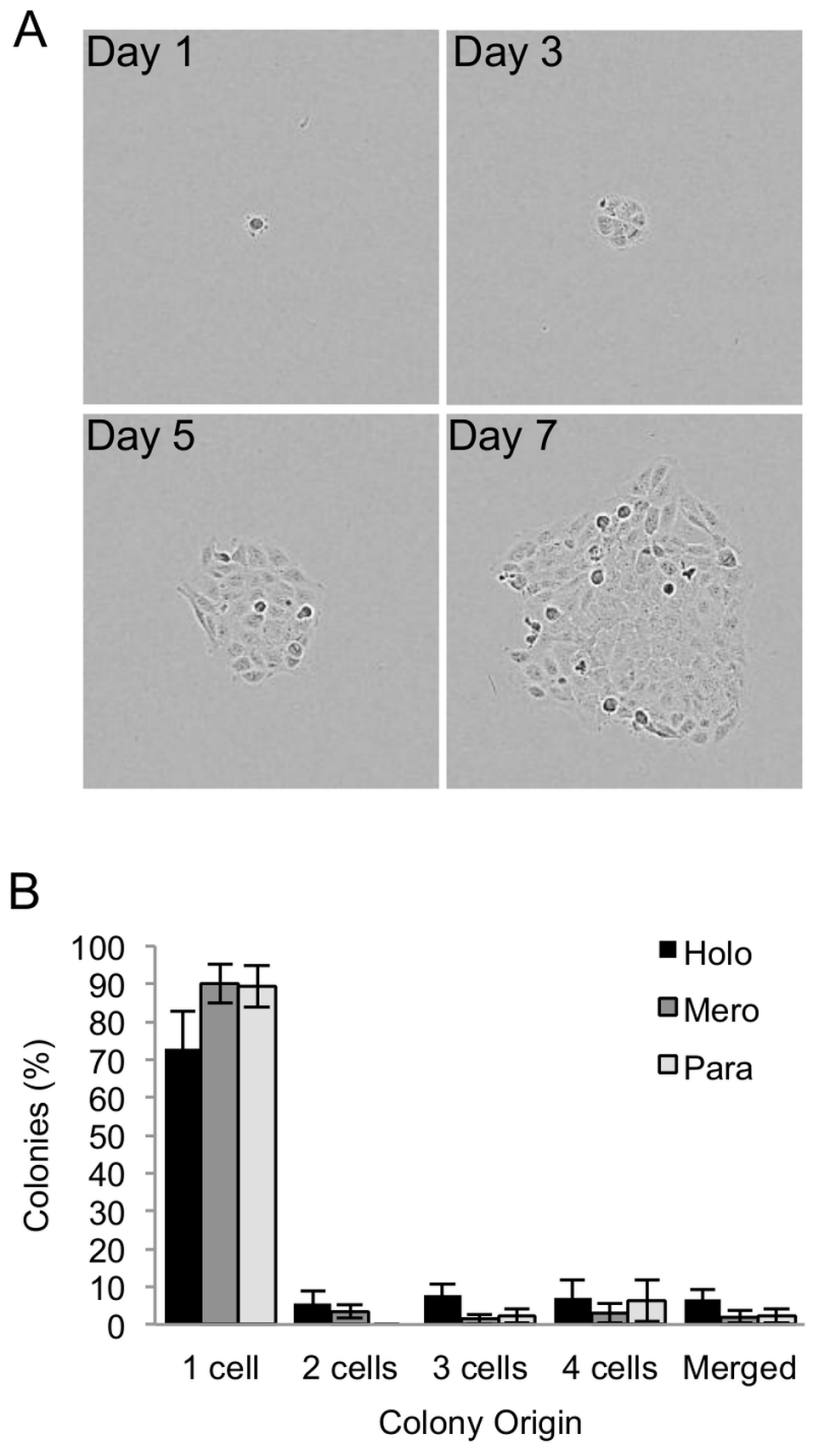
Single cell origin of DU145 colonies. (A) Formation of DU145 colonies from single cells was tracked by time lapse photography (Incucyte) every 4 hours from initial adherence for two weeks. (B) The number of colonies originating from 1 or more cells was determined. Colonies which were derived from a single cell upon initial adherence, but merged with other colonies were also determined. Results are displayed as mean ± S.E. from 5 experiments tracking 40 cells per experiment.

The ability to generate single cell suspensions for the purpose of cloning has not been previously checked systematically. The single cell origin of DU145 colonies was checked by time lapse photography using the Incucyte imaging system (Figure 5A). In each of five separate experiments, the origins of 30–50 colonies were determined by back-tracking. Although the majority of colonies were derived from single cells, it was observed that some colonies originated from multiple cells or from colonies which merged and by the time of fixation appeared to be one colony (Figure 5B). Of the holoclones, 72.9% ±9.8% were derived from single cells, compared to 89.5±5.2% and 89.2% ±5.5% of meroclone and paraclones.

## Discussion

The aim of this study was to test the hypothesis that holoclones and meroclones derived from a human cancer cell line differ only in the proportion of stem cells each contains. Both holoclones and meroclones contain cells which are highly proliferative, immortal, can self-renew and are serially tumorigenic, but in differing proportions. The evidence strongly supports the hypothesis and suggests that colony morphology cannot be used as a surrogate marker for a stem cell origin.

Tumours are believed to contain a hierarchy of cells headed by cancer stem cells (CSC) which can self-renew and differentiate to produce the multiple cell types observed within the cancer. To be considered a CSC, a cell must be able to self-renew, differentiate and be serially tumorigenic [30]. The results of this study show that both DU145 holoclones and meroclones contain cells with stem cell properties.

The first indication that DU145 meroclones contained self-renewing cells came from data demonstrating that both holoclones and meroclones can form all three secondary colony types, whereas paraclones have little self-renewal capacity. Self-renewal was further demonstrated by sphere formation by both holoclones and meroclones. The lower sphere forming efficiency and tendency of meroclones to form fewer holoclones suggests that meroclones contain a smaller proportion of self-renewing stem cells than holoclones.

Both holoclones and meroclones contained immortal cells which, when serially cloned, could be cultured for more than 100 divisions, whereas paraclones were terminal. Cancer stem cells are usually considered immortal and undergo many cell divisions to drive tumor growth and metastasis [31]. Both holoclones and meroclones were serially engrafted in nude mice. This assay is considered the gold standard for the identification of cancer stem cells [30]. Meroclones had a longer latency than holoclones and formed smaller tumours, again suggesting that meroclones contain fewer stem cells than holoclones.

CD44 and α2β1 integrin are markers that to enrich for a prostate cancer stem cell population [32]. α2β1 integrin was expressed at high levels in holoclones and meroclones but not paraclones, whilst CD44 was observed in holoclones and meroclones and at lower levels in paraclones. Another contrasting study has shown no difference between the growth rates of PC-3 cells in high cell density culture and showed no difference in CD44 and α2β1 expression [32], so these markers alone do not confirm CSC identity.

Holoclones and meroclones were positive for BMI-1, an oncogene suggested to play a role in stem cell self-renewal [33] and which has been previously shown to be up-regulated in pancreatic cancer cell holoclones [11]. The embryonic stem cell marker Oct-4 was observed in stem and transit-amplifying colonies, but not in paraclones, suggesting a role in self-renewal and differentiation [34]. Previous studies have shown that stem cell colony formation is controlled by factors involved in self-renewal, such as Nanog [35], telomerase [21] and microRNA miR-34a which controls CD44 expression [19].

A number of studies using prostate [14], [16], [17], [32] pancreatic [11] colo-rectal [36], breast [13], head and neck squamous cell caancer [12] and uveal melanoma [37] cancer cell lines have tried to validate the use of colony morphology as a surrogate marker to define colonies derived from stem cells, transit-amplifying cells and differentiating cells. The results are surprisingly disparate and are in contrast to our findings. All previous studies conclude that holoclones have a greater ability to be passaged in bulk culture [11], [17] or by serial cloning [11], [14], [16], [37] than paraclones, and that paraclones with a differentiated morphology have a very limited proliferative potential. In these studies, meroclones were either not grown [14], [16] or could only be propagated for about 3 months compared to more than 6 months for holoclones [17]. The ability of cells derived from meroclones to generate secondary holoclones was observed in one study, but few holoclones were formed from meroclones [11].

Previous studies have shown that only holoclones are tumorigenic *in vivo* [11], [17] or that holoclones form larger, faster growing tumors than paraclones [32], [36], [38]. Again, the majority of these studies only compared holoclones and paraclones. The ability of some paraclones to form tumours in some of these studies is paradoxical as it indicates that some paraclones contain stem cells.

Holoclones formed by the prostate cancer cell line PC-3 are highly tumorigenic, can be passaged long term and express the cancer stem cell markers α2β1+ CD44+ [17]. However, holoclones and meroclones are difficult to distinguish in cultures of PC-3 [16], [18].

It appears that colony morphology is a good predictor of stem cell origin in primary cultures derived from normal cells, but not cancer cell lines. In cancer, stem cell capacity may be shifted further down the cellular hierarchy towards differentiation, resulting in transit amplifying cells acquiring stem cell properties.

Using the Incucyte to track cell growth it was found that the majority of DU145 colonies are derived from single cells. A proportion of colonies were derived by the fusion of two colonies or from small clumps of cells. It is well known that some cell lines, such as LNCaP, are more prone to clumping and therefore produce fewer single cell derived colonies [39]. The results of this study show that the colony morphology of cancer cell lines cannot be used to distinguish an origin from stem or transit-amplifying cells. Holoclones and meroclones derived from the prostate cancer cell line DU145 differ only in the proportion of stem cells each contains.

## Supporting Information

Table S1 Antibodies used for analysis of DU145 colony marker expression.
(DOCX)
