# Supplementary material for: Clonogenicity: Holoclones and Meroclones Contain Stem Cells
Source: PLoS One. 2014 Feb 26;9(2):e89834. doi: 10.1371/journal.pone.0089834 (PMC3935944; doi:10.1371/journal.pone.0089834)
Supplement: Table S1 — Antibodies used for analysis of DU145 colony marker expression. (DOCX) [file pone.0089834.s001.docx]

Table S1. Antibodies used for analysis of DU145 colony marker expression.

| **Target** | **Primary Antibody (All Abcam unless stated)** | **Secondary Antibody (All Southern Biotech)** |
| --- | --- | --- |
| Ki67 (Proliferation) | Rabbit polyclonal 5µg/ml | Goat anti rabbit IgG FITC 4µg/ml |
| CK5 (Basal) | Mouse monoclonal clone XM26 5µg/ml | Goat anti mouse IgG1 FITC 4µg/ml |
| CK18 (Luminal) | Mouse monoclonal clone C-04 5µg/ml | Goat anti mouse IgG1 FITC 4µg/ml |
| α2β1 Integrin (stem cell) | Mouse monoclonal clone 16B4to 1µg/ml | Goat anti mouse IgG1 FITC4µg/ml |
| CD44 (stem cell) | Mouse monoclonal clone G44-26 (BD Biosciences) 5µg/ml | Goat anti mouse IgG2b FITC 4µg/ml |
| Oct-4 (stem cell) | Rabbit Polyclonal 4µg/ml | Goat anti rabbit IgG FITC 4µg/ml |
| BMI-1 (stem cell) | Rabbit Polyclonal 4µg/ml | Goat anti rabbit IgG FITC 4µg/ml |
